# Advanced Manufacturing-Driven Multiscale Nanostructured Metals: A Review

**DOI:** 10.1021/acs.nanolett.5c06441

**Published:** 2026-03-16

**Authors:** Xiangren Bai, Dongpeng Hua, Jian Lu

**Affiliations:** † Hongqiao Research Institute for Light Metal, Department of Mechanical Engineering, 53025 City University of Hong Kong, Hong Kong 999077, China; ‡ Hong Kong Branch of National Precious Metals Material Engineering Research Centre, City University of Hong Kong, Hong Kong 999077, China; § Research Department on Advanced Structural Materials and Additive Manufacturing, City University of Hong Kong Matter Science Research Institute (Futian), Shenzhen 518045, China

**Keywords:** Manufacturing, Nanostructure, Metal, Plastic Deformation, Deposition

## Abstract

The rapid evolution of next-generation advanced manufacturing technologies, exemplified by additive manufacturing, has drastically expanded the accessible compositional and processing windows for metallic materials. These advances actively drive the formation of far-from-equilibrium structures and open unprecedented opportunities to design multiscale nanostructured metals with exceptional structural and functional properties. In this mini-review, we examine how multiscale nanostructural design is driven by both top-down and bottom-up advanced manufacturing routes, elucidating the underlying thermodynamic, kinetic, and geometric driving forces. Representative approaches, including severe plastic deformation for top-down and physical/chemical vapor deposition for bottom-up, are analyzed to illustrate their distinctive roles in structure formation. The ensuing discussion highlights the strengthening and toughening mechanisms of these hierarchical structures from the perspectives of microscale dislocation regulation and mesoscopic deformation coordination. Finally, we summarize the performance advantages and application prospects of multiscale nanostructured metals as well as outline future pathways toward the deeper integration of multiscale structural design with emerging manufacturing technologies.

## Graphical abstract

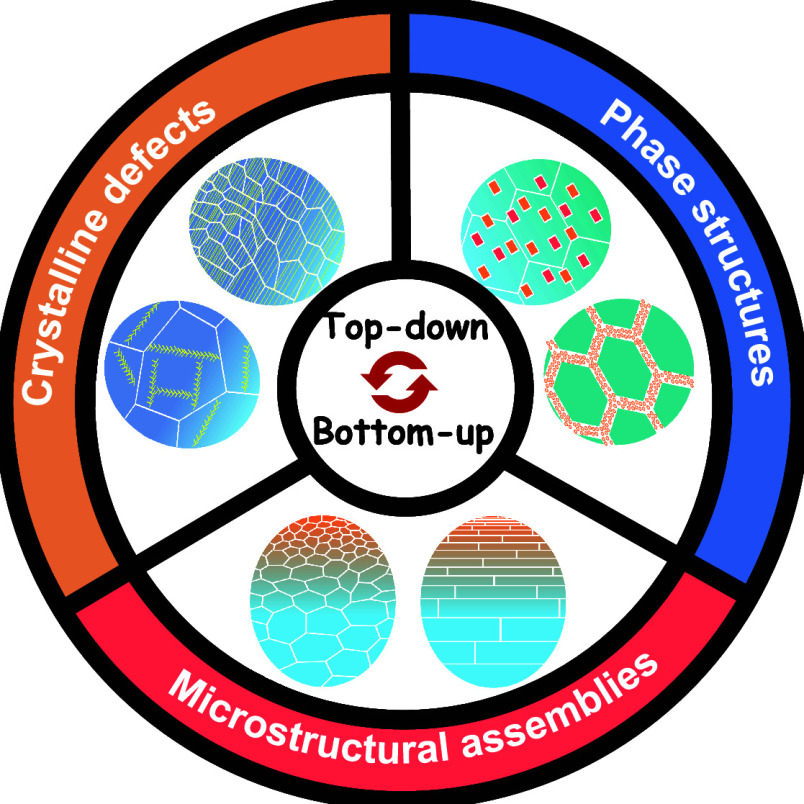

Effects intrinsic to characteristic structural scales place nanostructured metals at the forefront of modern materials research. The targeted design of hierarchical nanostructures has emerged as a promising paradigm for developing next-generation metallic systems with superior performance.^1,2^ These strategies aim to break long-standing trade-offs in metallic materials, such as the strength-ductility dilemma,^3,4^ strength-conductivity inversion,^5,6^ and size-effect limitations.^7^ Crucially, progress in this field has been catalyzed by advances in manufacturing technologies capable of imposing extreme, yet controllable, thermodynamic and kinetic conditions, thereby enabling precise control over structural features from atomic to macroscopic scales. Recent breakthroughs in laser-based additive manufacturing (AM) and other emerging processing routes exemplify this capability, dramatically expanding the accessible structural and compositional space.^8^

Nanostructured metallic materials derive their properties from structural features that span multiple hierarchical levels. From the perspective of plastic deformation, the resistance to plastic flow originates from the interactions between dislocations and structural obstacles on various scales. These interactions underpin several well-established strengthening mechanisms, including dislocation strengthening, dispersion strengthening, and grain boundary (GB) strengthening.^9^ Correspondingly, these mechanisms can be associated with three representative structural levels: (i) Crystalline defects, including dislocations, twins, and stacking faults (SFs), represent the most fundamental scale. Their type, density, and spatial distribution play critical roles in governing plastic flow and strain hardening. (ii) Nanoscale phase structures, such as short-range ordered (SRO) domains, precipitates, and dispersoids, introduce localized compositional and crystallographic heterogeneities. These features impede dislocation motion through elastic mismatch, modulus contrast, and chemical interactions. (iii) Microstructural assemblies, referring to grain-level features such as size distributions and crystallographic texture, determine how strain is partitioned across the material and strongly influence macroscopic mechanical behavior, particularly the strength-ductility balance.

The formation of nanoscale structures in metals typically requires locally nonequilibrium thermodynamic and kinetic conditions. These conditions, including extreme compositional gradients, steep temperature fields, and intense stress states, can promote the accumulation and stabilization of crystalline defects at densities characteristic of nanostructured materials.^10,11^ Recent advances in manufacturing technologies have significantly expanded the accessible structural design space beyond the limits imposed by conventional processing routes. Emerging techniques, such as severe plastic deformation (SPD)^12^ and physical or chemical deposition methods,^13,14^ allow for the stabilization of novel or metastable structures under far-from-equilibrium conditions. These developments not only enable the realization of theoretically ideal architectures but also facilitate the discovery of new structural configurations. Consequently, the paradigm of manufacturing-driven structural design and discovery is becoming an increasingly effective bridge between fundamental materials science and practical application.

In this mini-review, we discuss the thermodynamic-kinetic mechanisms that govern the formation of multiscale nanostructured metals through emerging manufacturing strategies (Figure 1). We outline accessible multiscale nanostructures, ranging from crystalline defects to phase structures to microstructural assemblies, and highlight how they confer distinctive mechanical properties. Finally, we clarify the strengthening and deformation mechanisms that govern structure–property relationships from the perspectives of microscale dislocation regulation and mesoscopic deformation coordination. We also discuss the application potential of multiscale nanostructured metals and conclude with perspectives on integrating multiscale design into next-generation manufacturing.

**Figure 1:**
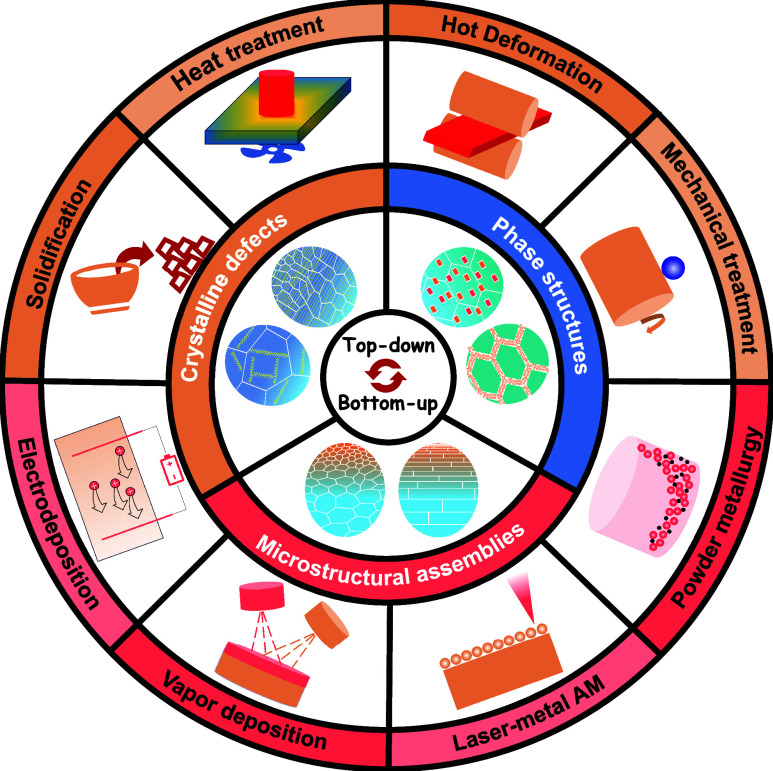
Typical top-down and bottom-up strategies to achieve multiscale nanostructures in metals.

## Top-Down Manufacturing Driven

Modern manufacturing technologies offer multiple pathways for inducing multiscale nanostructures in metallic materials, which can be broadly classified into top-down and bottom-up strategies. Top-down approaches emphasize the role of global thermodynamic constraints, wherein the macroscopic input of energy governs the evolution of thermal and stress fields, ultimately dictating phase transformation and microstructural evolution during processing. Under conventional processing conditions, the available means to impose significant external stress fields are limited, typically including quenching or modest levels of cold working.^15^ To achieve refined nanostructures, these methods must often be coupled with compositionally driven chemical forces that introduce localized driving forces for structural refinement. However, even with such combinations, it remains challenging to obtain ideal microstructural features and mechanical performance. A representative case is the precipitation process in aluminum (Al) alloys, where heat treatment or thermomechanical processing is employed to control the characteristics, size, morphology, and spatial distribution of precipitates for optimized strengthening.^16^ Nonetheless, inherent limitations in the nucleation of precipitates pose significant challenges. Achieving effective nucleation typically requires a high degree of supersaturation, which enhances the thermodynamic driving force and lowers the nucleation barrier, conditions that are generally accessible only at low temperatures.^17^ Conversely, atomic diffusion, which is essential for establishing sufficient compositional contrast between the matrix and the precipitate, is strongly dependent on elevated temperatures.^18^ Consequently, the commonly adopted peak-aging conditions represent a balance between thermodynamic and kinetic constraints. Yet, from the perspective of precipitation strengthening, the resultant precipitate density, size, and spatial distribution under such conditions often remain suboptimal relative to their theoretical potential.

To overcome the intrinsic conflict between thermodynamic requirements and kinetic constraints in conventional solid-state transformations and to enable the formation of finer and more diverse nanostructures in bulk metals, top-down strategies have increasingly focused on short-duration processing at low temperatures. These conditions are designed to suppress recovery processes that would otherwise degrade the desired microstructures. As a result, the high energy density necessary for the formation of nanostructures is primarily introduced through mechanical stress rather than thermal input. This approach has become a defining trend in the development of bulk nanostructured metals, where SPD at ambient or cryogenic temperatures is used to accumulate high densities of defects.^19^ Early implementations of this concept were realized through surface mechanical treatments, which introduced localized plastic deformation by imposing high strain rates in confined regions. Techniques, such as surface mechanical attrition treatment (SMAT),^20^ surface mechanical rolling,^21^ shot peening,^22^ and laser shock peening,^23^ have all been applied to create nanostructured surface layers. These methods typically achieve strain rates on the order of 10^2^–10^6^ s^–1^, which are sufficient to drive substantial microstructural refinement near the treated surface.^2^ The advancement of high-pressure processing technologies has further expanded the capabilities of top-down approaches. When combined with cryogenic environments such as liquid nitrogen cooling, methods including high-pressure torsion (HPT),^24^ equal-channel angular pressing (ECAP),^25^ and cyclic deformation^26^ have demonstrated the ability to introduce ultrahigh densities of deformation-induced defects throughout the bulk of the material rather than being limited to the surface. These improvements in processing methods not only enhance structural control but also enable the realization of previously inaccessible structural states. The evolution of such technologies continues to play a critical role in pushing the boundaries of materials design and discovery.

During SPD at ambient or cryogenic temperatures, high strain rates and large strain magnitudes lead to the prolific generation of dislocations. As the deformation proceeds, the associated flow stress increases significantly. Owing to the dominance of compressive stress states under high applied loads, the material resists fracture and maintains continuous plastic flow. These conditions promote a substantial reduction in the activation volume for dislocation motion and localize deformation, allowing even short dislocation segments or small atomic clusters to shear and subdivide the microstructure into high-density nanoscale domains.^27^ Concurrently, excess vacancies and interfaces are introduced during deformation. As the dislocation density increases, interactions among dislocations lead to the formation of dislocation cell walls. With progressive strain accumulation, these walls gradually evolve into low-angle subgrain boundaries and eventually transform into high-angle GBs.^28^ In materials with relatively low SF energy, such as certain face-centered cubic (FCC) alloys, the microstructure evolution is often accompanied by the formation of SFs and deformation twins. These planar defects interact with dislocations, facilitating the progressive subdivision of the coarse grains. Moreover, the presence of high concentrations of vacancies and nonequilibrium interfaces constrains short-range diffusion, thereby creating localized thermodynamic imbalances. These conditions provide a driving force for atomic rearrangements that result in the formation of new phase structures. Within such highly nanostructured environments, the compositional space of the alloy is also broadened. Solute solubility is frequently enhanced under these nonequilibrium conditions, particularly when the system is driven far from its thermodynamic ground state.^29^ The combination of lattice defects, interfacial structures, and altered phase compositions promotes the nanoscale engineering of structural features. These effects are further amplified in chemically complex alloys, where composition-induced chemical potentials assist in stabilizing metastable structures. Most critically, the low-temperature processing inherent to SPD effectively suppresses long-range atomic diffusion. This inhibition of thermal recovery stabilizes the newly formed nanostructures, enabling their retention under ambient conditions.

## Crystalline Defects

Under the influence of SPD, metallic materials can attain exceptionally high defect densities. Some studies have reported dislocation and planar defect densities on the order of 10^16^∼10^18^ m^–2^, far exceeding those achievable through conventional processing techniques.^30^ This increase primarily stems from enhanced dislocation activity, characterized by a reduced dynamic recovery or annihilation and a correspondingly elevated dislocation storage rate. More importantly, once strain hardening capacity is improved under such conditions, SFs and deformation twins are often activated under high applied stress. These features serve as secondary carriers of plastic deformation, introducing alternative pathways for accommodating strain beyond the traditional dislocation slip. Such phenomena result in a different defect pattern compared to that of conventional coarse-grained metals, dominated by line defects alone. For instance, recent work on AlCoCrFeNi high-entropy alloys (HEAs) subjected to low-temperature cyclic torsion has revealed the formation of nanoscale mosaic substructures composed of fine, closely spaced SFs oriented along different directions (Figure 2a–c).^30^ In these structures, the spacing between adjacent SFs or twin boundaries is typically less than 2 nm with an average segment length of approximately 20 nm. The formation of these novel defect configurations is attributed to the emission of SFs from dislocation cell walls accumulated during the early stages of deformation. These cell walls, enriched with dislocations, give rise to localized stress levels. At the same time, significant compositional fluctuations occur at these interfaces, altering the local SF energy and reducing the barrier for the partial dislocation emission. As deformation progresses and stress continues to rise, an increasing number of 2D fault interfaces propagate from the dislocation walls. These faults, activated on multiple {111} slip planes, interact to divide the original dislocation cell walls into arrays of parallel SF bundles. The newly generated SFs, along with their strong interactions with dislocation walls and isolated dislocations, act as additional, dynamically formed, and sustained sources of defects. These features enable the accelerated accumulation of both planar and line defects under sustained loading. Evidently, SPD offers one of the most direct and effective top-down pathways for engineering crystalline defects.^12^ The ability to manipulate such defect structures with precision is critical for the development and understanding of advanced nanostructured metals from a physical metallurgy perspective.

**Figure 2:**
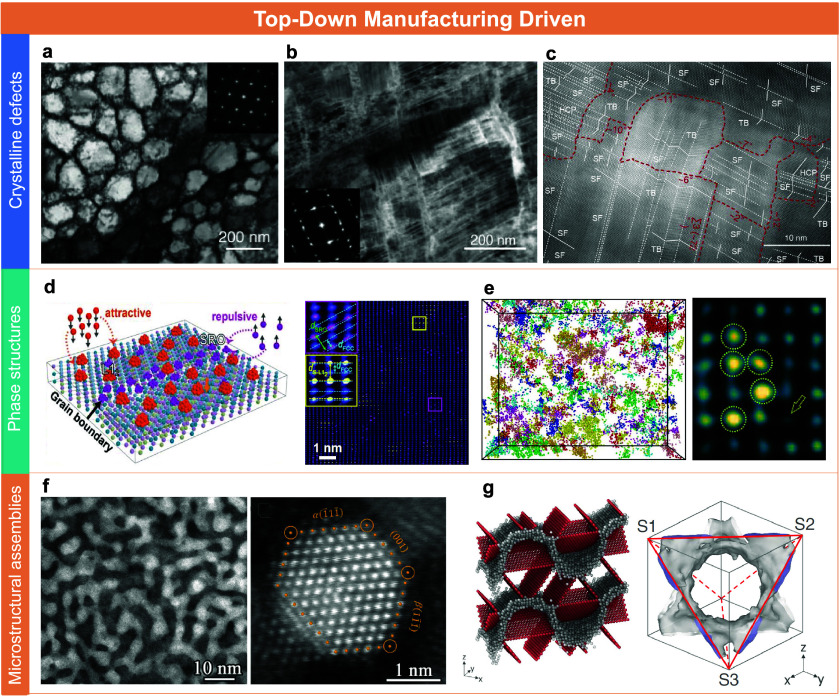
Multiscale nanostructures driven by top-down routes. (a–c) Typical microstructure of AlCoCrFeNi HEAs obtained by low-temperature cyclic torsion.^30^ Copyright 2019, reproduced with permission from The American Association for the Advancement of Science. (d) Illustration and TEM image of coupling SRO domains with supranano precipitates in CoVNiAl-based alloy.^31^ Copyright 2025, reproduced with permission from The American Association for the Advancement of Science. (e) Atom probe reconstructions and atomic-resolution HAADF-STEM image of the Mg–Cu clusters in Al alloys.^27^ Copyright 2019, reproduced with permission from The American Association for the Advancement of Science. (f) High-resolution TEM images of Schwarz-type crystalline networks in Cu.^32^ (g) Atomistic model and molecular dynamic simulations of Schwarz crystals.^32^ Copyright 2020, American Association for the Advancement of Science.

## Phase Structures

Beyond the manipulation of crystalline defects, SPD also plays a crucial role in enabling and driving the formation of novel nanoscale phase structures, including ultrahigh-density precipitates and SRO domains. These features stem from a delicate balance between atomic diffusion kinetics and nanoscale thermodynamic constraints, both of which are actively modulated under SPD conditions. In HEAs, for example, SPD combined with subsequent thermal treatment can drive the coupling of SRO domains with ultrafine precipitates, thereby enhancing both strength and ductility. A representative microstructure consists of coherent L1_2_ supernano precipitates, typically 0.5–4 nm in size. They are embedded within a CoVNiAl-based FCC matrix, alongside SRO domains segregated near GBs (Figure 2d).^31^ The formation of this dual structure is driven by local chemical fluctuations that promote the development of ordered atomic configurations. Initially, rapid quenching suppresses long-range diffusion and preserves compositional heterogeneities. Subsequent thermal- or deformation-induced atomic rearrangement facilitates the nucleation of ultrafine precipitates in regions enriched with short-range order. The combined effects of interfacial energy, lattice distortion, and vacancy-mediated diffusion ultimately stabilize this coupled SRO-precipitate configuration. Such a mechanism is particularly significant for Al alloys that rely heavily on high-density precipitates for strengthening (Figure 2e).^27^ Under SPD processing at room temperature, precipitate coarsening, typically observed in conventional thermal aging, is effectively avoided. Instead, SPD promotes both intra- and intergranular compositional structuring. This behavior stems from the high density of dislocation activity induced by alternating external stresses during processing. The bowing and tangling of dislocations generate an abundance of vacancies, which in turn enhance the solute diffusivity even at low temperatures. As a result, solute atoms can migrate and locally aggregate, forming high-density solute clusters. Importantly, these clusters remain extremely fine due to limited long-range diffusion and additional shear effects imparted by dislocation motion. Their increasing density, coupled with a constrained size, leads to a highly refined and stable precipitate network. Moreover, because plastic deformation typically initiates in regions with lower initial resistance, the evolution of the microstructure during SPD proceeds in a spatially balanced manner. This ensures that newly formed defects tend to populate previously defect-sparse zones, thereby homogenizing the distribution of local strength and chemical composition.^27^ The outcome is a uniformly refined microstructure, which provides a highly favorable platform for the subsequent phase design and performance optimization.

## Microstructural Assemblies

At the grain-assembly scale, recent studies on Schwarz-type crystalline networks have further highlighted the critical role of processing in enabling extreme microstructural refinement (Figure 2f).^32^ Via combining surface mechanical grinding treatment (SMGT) with HPT, such structures, characterized by an ideal ultrafine grain size of approximately 5 nm, have been realized in various metallic systems. Notably, these nanograined configurations exhibit remarkable thermal stability, maintaining their fine grain size even at temperatures near the melting point. The conceptual origin of this structure lies in the hypothesis that nanocrystalline metals may transition into more stable configurations as the grain size approaches the extreme lower bound (Figure 2g). This hypothesis has been validated through advanced SPD routes that provide sufficient mechanical energy to progressively transform initially unstable crystalline defects into a stabilized network of minimum-interface configurations. In this state, GBs are predominantly composed of Σ3 (111) coherent twin boundaries, which serve to constrain interfacial motion and inhibit grain coarsening. The evolution of such ordered architectures arises from a combination of processing-driven kinetic forces and interface-governed local thermodynamic equilibria. During deformation, excessive dislocations and high-angle boundaries are gradually eliminated via atomistic rearrangements and GB migration, ultimately forming a periodic and low-energy interfacial network. From a crystallographic perspective, the formation of this structure is likely mediated by deformation-induced twinning, which is difficult to achieve through conventional means. Thus, this example strongly illustrates the role of advanced processing in not only tailoring known structures but also in discovering previously inaccessible nanostructured configurations.

## Bottom-Up Manufacturing Driven

In contrast to top-down strategies, bottom-up approaches emphasize the construction of complex structures starting from atomic units, clusters, or particles through locally driven, nonequilibrium kinetic processes. The central principle of this strategy lies not in imposing global thermodynamic constraints but in precisely regulating local growth conditions during atomic deposition to direct the ordered evolution of microstructures. This framework encompasses a broad range of physical or chemical deposition techniques, including physical vapor deposition (PVD),^33^ chemical vapor deposition (CVD),^34^ electrochemical deposition,^35^ atomic layer deposition (ALD),^36^ and powder-based AM methods for metals.^8^ Despite differences in processing scale and complexity, these techniques share a common underlying logic in which structures are built layer by layer during deposition rather than being derived from pre-existing bulk material. This decouples microstructural development from traditional equilibrium constraints, thus, expanding freedom to explore and control structural evolution. As such, bottom-up strategies provide exceptional tunability in processing and have become a powerful driver for the discovery of novel nanostructures.

Among various bottom-up approaches, atomic-scale deposition techniques offer the best control over structural features. PVD, exemplified by magnetron sputtering, enables precise tuning of deposition kinetics through adjusting parameters such as power input, working pressure, and substrate temperature. Under nonequilibrium conditions, this facilitates exceptionally high nucleation rates and the formation of ultrafine grains, and allows for the stabilization of amorphous structures.^37^ Furthermore, limited surface diffusion and localized supersaturation during deposition can promote the formation of high-density defect structures, including SFs, nanotwins, and nonequilibrium interfaces. By employing alloyed targets or reactive gas environments, compositional control can be extended to the subnanometer scale, enabling the fabrication of multilayer films. These microstructural features arise not as incidental byproducts but as direct outcomes of the interplay between interfacial energy, compositional gradients, and energetic inputs during deposition. They highlight the intrinsic capability of bottom-up strategies to direct structural evolution through localized kinetic pathways. The formation of such microstructural features arises from the synergistic effects among interfacial energy regulation, local compositional gradients, and kinetic energy input during deposition.^38^ However, despite offering exceptional control over microstructural features, atomic-scale deposition techniques generally suffer from limited throughput, making them more suitable for thin films and applications for which structural precision is paramount. To extend the applicability of bottom-up strategies to larger length scales, metal AM has emerged as a powerful platform in recent years. Techniques such as laser powder bed fusion (LPBF), directed energy deposition (DED), and wire arc additive manufacturing (WAAM) utilize localized energy sources to rapidly melt and solidify metallic powders, enabling layer-by-layer construction at the mesoscale.^8,38^ Although initially developed for geometric customization, metal AM has evolved into a versatile tool for fabricating nonequilibrium microstructures due to its ability to generate extremely high solidification rates, steep thermal gradients, and complex thermal cycling conditions.^39^ These features promote the formation of metastable architectures such as cellular subgrains, solute-enriched boundaries, supersaturated solid solutions, etc. Additionally, the layerwise nature of AM and its localized thermal control enable the creation of microstructural gradients and texture selectivity, allowing the formation of gradient nanostructures unattainable by conventional processing. By tailoring scanning strategies and process parameters, we can also construct functionally graded materials within a single component.

Importantly, bottom-up approaches offer a high degree of modularity and tunability, enabling the redesign of complex multicomponent systems that were previously constrained by segregation tendencies or processing limitations. This flexibility is particularly valuable in alloy systems with significant compositional complexity such as HEAs. The careful control of deposition parameters allows for the deliberate introduction of nonequilibrium chemical heterogeneity, the coupling of short-range order with nanoscale precipitation, and the stabilization of unique defect-phase architectures. These features contribute to an exceptional combination of mechanical strength and ductility, unattainable via conventional alloying and processing routes. Notably, many of these structures fall outside the predictions of traditional equilibrium phase diagrams, highlighting the critical role of processing-driven kinetic pathways in unlocking new microstructural regimes. Furthermore, bottom-up fabrication has facilitated the discovery of structural building blocks that are rarely accessible through thermally dominated routes. These features often emerge from local kinetic instabilities during deposition or solidification and rely on the interplay between the processing variables and interfacial energetics. Taken together, bottom-up strategies extend the design space for structural metals by decoupling the development of microstructures from traditional thermodynamic constraints. Through precise regulation of energy delivery, chemical flux, and interfacial evolution, they enable the stabilization of complex, hierarchical, and nonequilibrium nanostructures across multiple length scales.

## Crystalline Defects

Bottom-up physical and chemical deposition techniques have significantly broadened the scope of defect engineering in metallic materials and have become a key focus in nanostructured metals. These methods enable the formation of unconventional defect structures closely associated with an enhanced mechanical performance. Electrodeposition is a well-studied case that demonstrates the ability to introduce dense nanotwin networks in a range of metals such as copper, nickel, and cobalt (Figure 3a, b).^35,40^ By adjustment of deposition parameters, including current density and pulse timing, the kinetics of atom deposition can be carefully tailored. This control allows the twin spacing to be tuned from several micrometers down to just a few nanometers. The formation of such features is driven by the nonequilibrium nature of the manufacturing process. Rapid atomic deposition provides a strong thermodynamic driving force for nucleation, while limiting the time available for recovery, which favors the statistical formation of coherent twin boundaries over high-angle GBs. Building on this atomic-scale control, metal-laser-based additive manufacturing introduces additional complexity to bottom-up fabrication by generating highly localized and dynamic thermal fields during processing. These include steep temperature gradients, rapid solidification rates, intense residual stresses, and transient compositional nonuniformities. These conditions strongly promote the development of nonequilibrium defect structures. In particular, LPBF has enabled the appearance of SFs and twin boundaries in Al alloys, despite their intrinsically high SF energy (Figure 3c).^41^ The combination of rapid solidification, repeated thermal cycling, and residual stress accumulation provides favorable conditions for stabilizing rare phases, such as 9R structures and SF configurations. It is important to note that the formation of these defect structures also critically depends on specific alloy chemistries, particularly the use of high concentrations of elements such as Sc, Zr, Mn, and Mg. These alloying additions can significantly lower the local SF energy and facilitate defect nucleation. However, incorporating such high concentrations is often impractical in conventional processing due to solubility limits and phase stability issues. In contrast, the rapid solidification and unique thermal profiles inherent to AM enable these compositions to be successfully retained, thereby promoting the formation of otherwise inaccessible defect configurations. In addition to planar defects, AM processes often produce nanoscale dislocation cell structures during solidification (Figure 3d).^42^ These cells commonly exhibit solute segregation at cell boundaries and are accompanied by the formation of nanoscale precipitates. This hierarchical defect configuration contributes to enhanced strain hardening and damage resistance. As a result, AM-fabricated metals can achieve an exceptional combination of strength and fracture toughness, outperforming many conventionally processed alloys. These observations highlight the central role of process-driven defect engineering in bottom-up manufacturing strategies.

**Figure 3:**
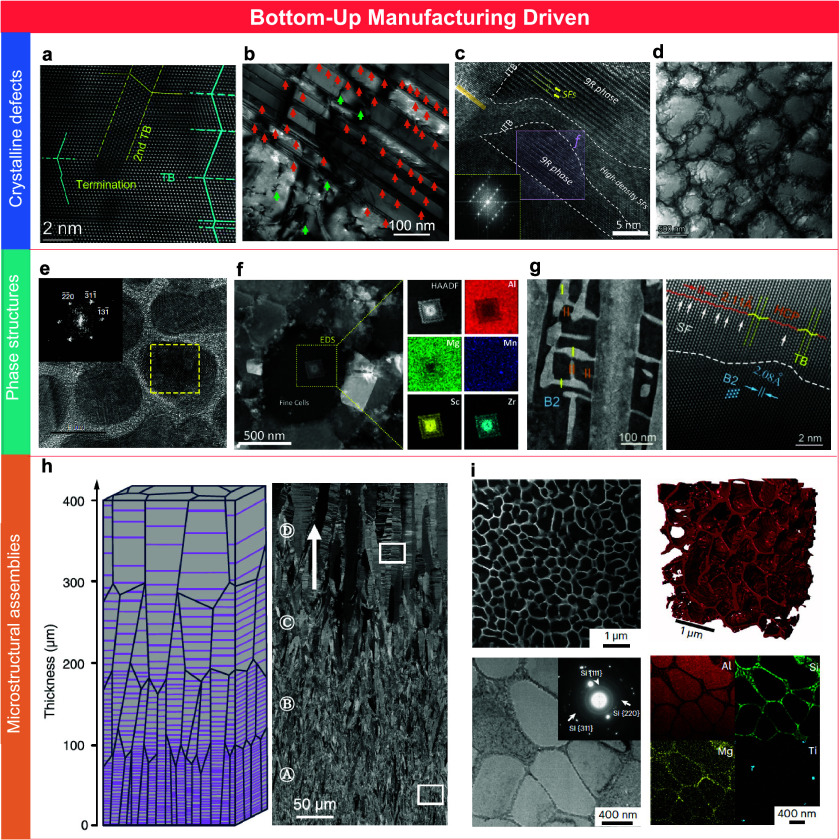
Multiscale nanostructures driven by bottom-up routes. (a) Typical atomic microstructure of as-deposited nanotwinned Ni with an extremely fine twin thickness.^35^ Copyright 2021, reproduced with permission from The American Association for the Advancement of Science. (b) TEM image of the nanotwins and bundles of concentrated dislocations in electrodeposited Cu.^40^ Copyright 2018, reproduced with permission from The American Association for the Advancement of Science. (c) TEM image of the nanotwins and different 9R phase regions in LPBFed Al alloys.^41^ Copyright 2024, reproduced with permission from Elsevier. (d) TEM image of the dislocation cell structures in LPBFed Ti alloys.^42^ Copyright 2026, reproduced with permission from Springer Nature. (e) HRTEM image of crystalline–amorphous nanocomposite structure in PVD magnesium alloy.^43^ Copyright 2017, reproduced with permission from Springer Nature. (f) HAADF-STEM image and corresponding EDS map of the nano intermetallic compounds within the grain in AMed Al alloys.^41^ Copyright 2024, reproduced with permission from Elsevier. (g) Nanotwinned precipitates in AMed AlCoCrFeNi-based HEA.^39^ Copyright 2025, reproduced with permission from Elsevier. (h) Schematic and SEM observation of the gradient grain structure in electrodeposited Cu.^40^ Copyright 2018, reproduced with permission from The American Association for the Advancement of Science. (i) Continuous eutectic silicon networks in AlSi10Mg alloys fabricated via LPBF.^44^ Copyright 2023, reproduced with permission from Springer Nature.

### Phase Structures

Similar to the formation of defect patterns, the bottom-up assembly of metal atoms can be kinetically and locally thermodynamically driven to generate novel phase structures. For instance, PVD techniques have been used to fabricate crystalline–amorphous nanocomposite structures (Figure 3e).^43^ Under magnetron sputtering conditions, rapid quenching suppresses long-range diffusion and stabilizes supersaturated solid solutions, while nonequilibrium vacancy flux and interface segregation promote the concurrent nucleation of crystalline domains and amorphous regions. The combined effect of kinetic constraints and interfacial energy leads to ultrafine biphasic structures where nanocrystalline grains are embedded within an amorphous matrix, yielding materials with strengths approaching theoretical limits. More recently, the advancement of metal AM has enabled the full development of such nanoscale phase structures in large-scale, industrially relevant components. A representative breakthrough lies in the development of heat-resistant Al alloys. This is enabled by the significantly enhanced solubility of traditionally low-solubility elements such as Fe, Ti, Zr, and Ni under AM conditions, which facilitates the in situ formation of nanoscale, thermally stable intermetallic compounds (Figure 3f).^41^ This represents one of the most emblematic examples of AM-driven alloy discovery. The long-standing concept, enhancing thermal stability in Al alloys through stable nanoscale intermetallics, has only recently become technically feasible due to the nonequilibrium nature of AM. When combined with the emerging design space of multiprincipal element alloys, AM techniques have enabled even more diverse nanophase configurations. For example, in an AlCoCrFeNi-based HEA initially dominated by a metastable B2 matrix, postdeposition annealing after laser AM induces a hierarchical precipitation structure composed of FCC nanodomains with internal nanotwins (Figure 3g).^39^ This transformation is governed by a two-step mechanism involving local compositional fluctuations and internal stress redistribution, both amplified by the thermal conditions inherent to AM. By enabling access to nonclassical phase sequences, the development of bottom-up manufacturing technologies provides a powerful driving force for realizing metastable pathways inaccessible to conventional thermodynamic processing.

## Microstructural Assemblies

As the fundamental structural and functional units of metallic matrices, grain-scale structures are directly influenced by the layer-by-layer deposition nature of the bottom-up fabrication. By controlling nucleation, growth thermodynamics, interfacial kinetics, and geometric confinement, it is now possible to tailor features such as grain size gradients, crystallographic texture, and hierarchical structures with high precision. For instance, by modulating the electrolyte temperature during direct current electrodeposition, researchers have fabricated gradient nanograined copper that exhibits dual gradients in both grain size and twin thickness, which represents a hallmark example of the emerging class of heterostructured materials (Figure 3h).^40^ Similarly, metal AM offers unique flexibility in microstructural design due to its process complexity and parameter richness. A variety of parameters including laser source characteristics, beam shape, scanning strategy, substrate heating, and auxiliary fields can all significantly influence the shape and thermal profile of the melt pool. This in turn governs the solidification dynamics and final grain architecture, enabling control over the grain size, morphology, and texture beyond equilibrium limitations. A representative case is the formation of continuous eutectic silicon networks in AlSi10Mg alloys fabricated via LPBF (Figure 3i).^44^ Unlike conventional casting, which typically produces coarse and discontinuous Si particles, LPBF generates a fine, interconnected, honeycomb-like Si framework embedded in the Al matrix. This morphology results from the coupled eutectic growth at the solidification front, facilitated by the rapid solidification and localized thermal cycling intrinsic to AM. The simultaneous nucleation of the Al and Si phases, along with suppressed grain coarsening, is key to forming this metastable structure. The resulting microstructure significantly enhances fatigue resistance by mitigating stress concentrations and delaying crack initiation. Clearly, bottom-up strategies enable precise spatial and kinetic control over grain-scale assemblies, unlocking metastable architectures that are inaccessible via conventional processing and crucial for next-generation performance optimization.

## Multiscale Strengthening and Synergetic Deformation Mechanisms

Previous discussions have elucidated how advanced manufacturing processes leverage unique thermodynamic and kinetic nonequilibrium regulations to achieve the precision architecture of multiscale nanostructures. This leap has transformed the fundamental structural units within materials from passive responders to deformation into intelligent units capable of actively modulating the deformation behavior. Through multiscale physical coupling, these nanostructures collectively endow materials with exceptional mechanical properties.^31,39,41,45–48^ At the microscopic scale, crystal defects and nanophases enable the precise regulation of dislocation nucleation, mobility, and storage, thereby synergistically enhancing strength-ductility combinations and work-hardening capacity.^31,45^ At the mesoscopic scale, the deformation incompatibility between “soft” and “hard” microdomains triggers heterogeneous-deformation-induced (HDI) strengthening. The resulting HDI stress maintains a high work-hardening rate, providing an additional strengthening and work-hardening contribution.^48^ Furthermore, the synergy of multiphase microstructures endows the material with the ability to suppress strain localization and blunt crack propagation.^49^ This mechanistic coupling across microscopic to mesoscopic scales constitutes the physical foundation for metallic materials manufactured through advanced techniques to transcend traditional performance limits. This section systematically elucidates the multiscale strengthening and synergetic deformation mechanisms.

## Dislocation Behavior Regulation at the Microscopic Scale

At the microscopic scale, the mechanical properties of materials are fundamentally governed by the nucleation, mobility, and storage behaviors of dislocations. Advanced manufacturing empowers researchers with the capability for on-demand design of multiscale nanostructures, including crystal defects, SRO, and nano secondary phases. This enables their synergistic control over dislocation behavior, thereby achieving a coordinated enhancement of both strength and toughness. This control manifests in three primary aspects: (i) regulating the dislocation nucleation threshold and spatial distribution via interfaces, chemical heterogeneity, and local stress concentrations, (ii) modulating dislocation slip modes and resistance through fluctuating lattice energy landscapes and nanophase distributions, and (iii) providing a dynamic platform for dislocation accumulation and multiple reactions through the topology of defects and the distribution of nanophases.

Dislocation nucleation controls the onset of plastic deformation, making the nature and spatial distribution of dislocation sources critical for the strength-ductility synergy. In conventional materials, dislocation nucleation typically occurs stochastically at grain boundaries or pre-existing defects.^50^ However, nanostructures introduced via advanced manufacturing can transform this process from random spontaneity to oriented regulation. Taking alloys fabricated by nonequilibrium processes such as LPBF as an example, the ultrafast cooling rates and complex thermal histories within the melt pool promote the formation of high-density dislocation cellular structures.^51^ The cell walls can evolve into sustainable sources for dislocation emission during subsequent deformation (Figure 4a), thus helping to maintain high strength while mitigating early stage cracking caused by the scarcity of dislocation sources. The SRO generated by nonequilibrium processes constitutes an atomic-scale chemical heterogeneity that also significantly modulates dislocation nucleation behavior. Spatial fluctuations in local bonding states and lattice stiffness facilitate the heterogeneous nucleation of dislocations at specific chemical environments.^52,53^ This promotes the activation of multiple slip systems and can induce the initiation of hierarchical twinning, contributing to subsequent work hardening. As these chemical clusters grow further, they can evolve into nanoprecipitates with well-defined interfaces with the matrix. Due to lattice mismatch and associated stress concentrations, these interfaces can act as highly efficient dislocation sources, allowing localized stresses to be relieved via plastic flow, thereby substantially reducing the risk of early stage cracking.^54^ Consequently, by constructing 3D dislocation cell networks and introducing SRO domains and nanoprecipitates, nonequilibrium processes shift dislocation sources from limited grain boundaries/defects to controllable structural units distributed throughout the grain interior, establishing the physical foundation for stable plastic deformation in high-strength materials.

**Figure 4:**
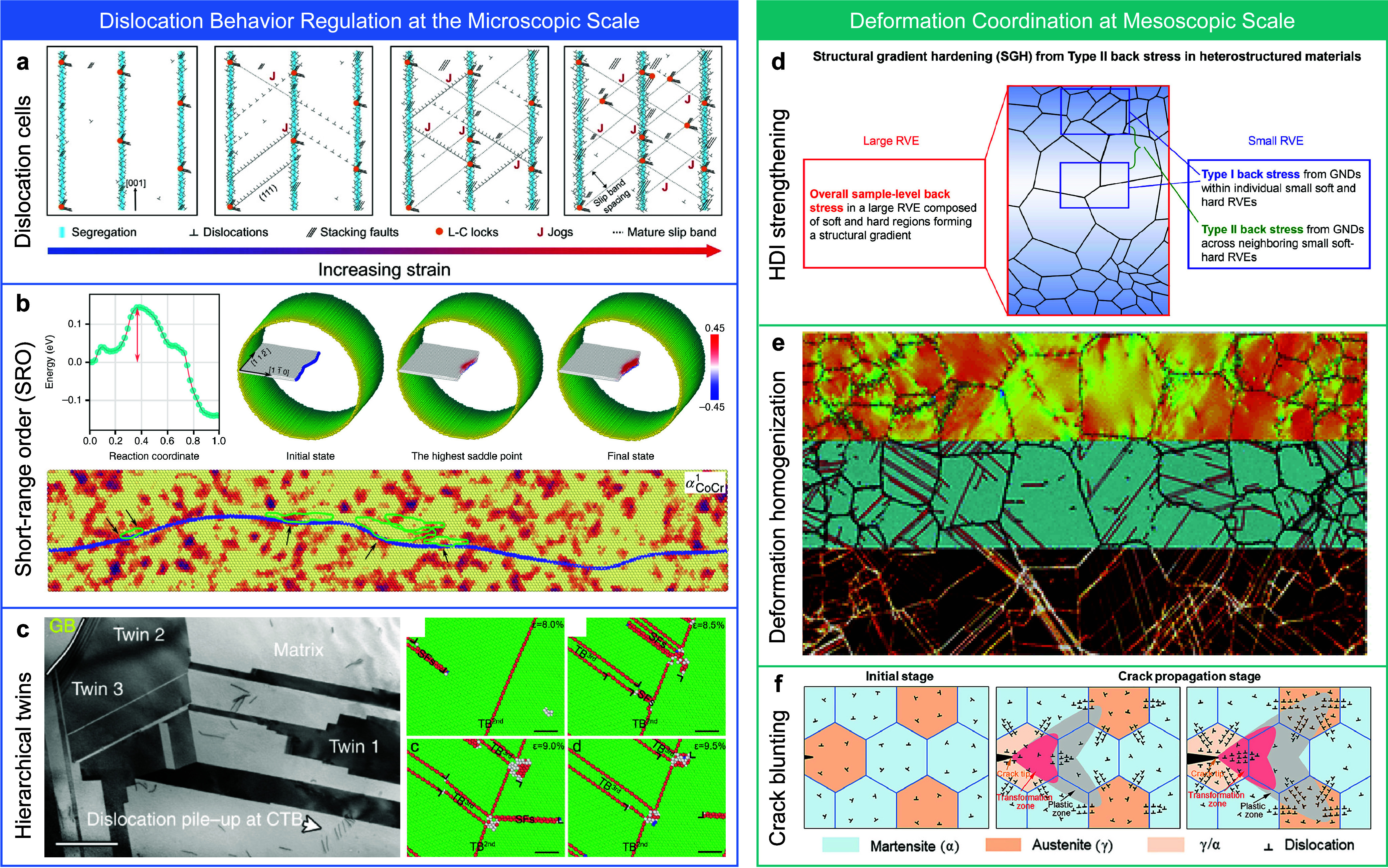
Multiscale strengthening and synergetic deformation mechanisms. (a) Schematics of the dislocation behavior regulated by segregation-dislocation cell structures.^51^ Copyright 2025, reproduced with permission from Springer Nature. (b) Fluctuating energy landscape and “trapping-detrapping” intermittent glide behavior induced by SRO.^55^ Copyright 2019, reproduced with permission from Springer Nature. (c) (Left) Bright-field TEM image showing a hierarchical twinning architecture in CrCoNi alloy.^61^ Copyright 2017, reproduced with permission from Springer Nature. (Right) Influence of hierarchical twinning on dislocation glide revealed by md simulations.^62^ Copyright 2020, adapted and reproduced with permission from Elsevier. (d) Schematic of a large representative volume element (RVE) composed of soft and hard domains in heterostructured materials.^65^ Copyright 2026, reproduced with permission from Elsevier. (e) Deformation homogenization in gradient structures, showing a smooth stress and strain gradient from nanocrystalline surface layers to coarse-grained interior regions.^69^ Copyright 2020, reproduced with permission from American Chemical Society. (f) Schematic of the toughening effect in heterogeneous dual-phase steel, driven by dislocation transmission across dual-phase interfaces and dislocation absorption at crack sites.^49^ Copyright 2025, reproduced with permission from National Academy of Science.

Following the establishment of abundant and controllable dislocation sources, the capacity for dislocation glide within the lattice emerges as the central determinant of the strength and ductility. In the multiscale nanostructures engineered by advanced manufacturing, dislocation motion is rendered substantially more complex. At the atomic scale, SRO creates a fluctuating energy landscape within the lattice.^55^ As dislocations glide, they are temporarily pinned at local energy saddle points. However, the pinning forces generated by SRO are relatively weak, permitting dislocations to advance upon accumulation of sufficient energy (Figure 4b). This results in a distinctive “trapping-detrapping” intermittent glide behavior that enhances strength while preserving ductility. At a larger scale, nanoprecipitates introduce further resistance to dislocation motion, with the interaction mechanism critically dependent on their size, spacing, and coherency. Nanoprecipitates maintaining a coherent relationship with the matrix exhibit a pinning and shearing mechanism analogous to, but more potent than, that of SRO.^56^ As coherency diminishes, nanoprecipitates exert stronger pinning effects, thereby conferring ultrahigh strength upon the material.^57^ When precipitate size is small and interparticle spacing is limited, dislocations tend to bow and loop between them, characteristic of the Orowan bypassing mechanism.^58^ As particle size and stiffness increase further, local stress concentrations intensify, making it difficult for dislocations to shear or bypass, which often leads to dislocation pile-ups.^59^ It is noteworthy that high densities of 2D planar defects (such as cell walls, stacking faults, twins, grain boundaries, etc.) also act as potent barriers to dislocation slip, where the resistance strength and characteristic dimensions follow the classical Hall-Petch relationship.^60^ Consequently, a variety of nanostructure units, ranging from SRO and nanoprecipitates to 2D planar defects, constitute a hierarchical system of dislocation obstacles, which can achieve a synergetic regulation of strength and plasticity by precisely modulating dislocation slip modes and resistance.

The dynamic storage of dislocations fundamentally dictates the work-hardening behavior and ductility of materials. In conventional alloys, 2D planar defects impede dislocation glide, leading to dislocation pile-ups.^60^ This static mode of dislocation storage, dominated by simple accumulation, is often accompanied by pronounced local stress concentrations, which can readily induce premature necking or crack nucleation in high-strength alloys. In contrast, the defect topologies and spatial distributions of nanophases engineered by advanced manufacturing provide unique geometric constraints and reaction platforms for complex dislocation interactions and dynamic storage. This architecture enables the maintenance of high dislocation density storage while concurrently introducing multipath escape channels for dislocation climb or cross-slip. Consequently, dislocations can continuously participate in plastic deformation within nanoconfined spaces, sustaining a high work-hardening rate even under elevated stress levels. Hierarchical nanotwins with multiple symmetries serve as the quintessential example. These three-dimensional twin networks not only act as robust obstacles to dislocation motion but also provide pathways for dislocation climb and cross-slip (Figure 4c).^61,62^ This allows piled-up dislocations to be redistributed along the twinning network, thereby preventing the rapid accumulation of localized damage. Similarly, the 3D dislocation cell structures inherent to additively manufactured materials promote the activation of multiple slip systems, leading to the formation of high densities of Lomer-Cottrell locks and dislocation jogs (Figure 4a). This configuration not only stores dislocations effectively but also enhances the capacity for cross-slip. Furthermore, nanophases augment the dislocation storage density through distinct mechanisms. On one hand, nanophases extend the residence time of gliding dislocations within localized regions, increasing the probability of interaction and entanglement with other dislocations, thereby increasing dislocation density.^45^ On the other hand, as dislocations cut or bypass through nanophases, they frequently generate new dislocations or Orowan loops at the precipitate interfaces, further driving up the dislocation density.^58^

In summary, the modulation of dislocation behavior at the microscopic scale serves as the cornerstone of multiscale strengthening. By precisely tailoring nanoscopic crystal defects and phase architectures, advanced manufacturing enables the synergetic control of dislocation nucleation, mobility, and storage, thereby offering unprecedented opportunities to transcend traditional strength-ductility trade-offs. Representative recent breakthroughs underscore this principle. For instance, by collaborative design of grain-boundary SRO and intragranular nanoprecipitates, we yielded an ultrastrong alloy with a 2.6 GPa yield strength and 10% fracture strain.^31^ Furthermore, work by Lu et al. demonstrated that the codesign of SRO and ordered nanoprecipitates can achieve exceptional strength-ductility synergy even at cryogenic temperatures (87 K).^45^ These advancements underscore the potential of leveraging multiscale nanostructures in pushing the boundaries of metallic material performance.

## Deformation Coordination at the Mesoscopic Scale

When the aforementioned nanostructures are organized by advanced manufacturing into mesoscopic heterogeneous architectures, they give rise to HDI strengthening and hardening, which transcend the contributions of microscopic dislocation behaviors. These architectures are collectively termed heterostructured materials, with prominent examples including bimodal grain structures (a mixture of nano- and microscale grains), gradient structures (gradual size variation), brick-and-mortar layered configurations (staggered interfaces), and bioinspired structures (e.g., bamboo-like or nacre-like structures, etc.).^63^ Despite their diverse morphologies, the essence of their strengthening and hardening stems from the deformation coordination and damage regulation effects between “soft” and “hard” domains with distinct mechanical properties.

Under external loading, soft and hard domains exhibit disparate plastic responses due to their yield strength differentials. During the initial stages of plastic deformation, soft domains with lower flow stress (e.g., coarse-grained regions or soft phases) yield first, while high-strength hard domains (e.g., ultrafine-grained regions or hard phases) remain elastic. This strain incompatibility generates significant strain gradients at domain boundaries.^64^ It is important to clarify that the interface between soft and hard domains at the mesoscale is not a conventional sharp 2D boundary. Instead, it constitutes a spatially extended heterogeneous boundary-affected region (HBAR), which includes a soft-domain side (HBAR-S) and a hard-domain side (HBAR-H), and can span multiple grains. To maintain macroscopic strain continuity, a high density of geometrically necessary dislocations (GNDs) must be generated in HBAR-S to accommodate the strain gradient. The accumulation of GNDs in HBAR-S induces a back stress within the soft domain, thereby strengthening it. In the hard domain, stress concentration manifests as a forward stress, which promotes earlier yielding. The asymmetric spatial attenuation of the back and forward stresses results in a net strengthening effect, known as HDI stress.^64^ The presence of HDI stress not only enhances yield strength but also continues to accumulate with an ongoing deformation, leading to sustained work hardening. Notably, the accumulation of GNDs in the HBAR-S operates on a fundamentally different scale compared with the pile-up of gliding dislocations at planar defects such as grain boundaries. While dislocation pile-ups at planar defects also generate back stress (referred to as Type I back stress), its contribution to strengthening is relatively limited.^64,65^ The primary strengthening mechanism in such cases remains obstruction of the dislocation glide by the interface, as described by classical Hall-Petch strengthening. In contrast, the accumulation of GNDs in the HBAR-S produces a long-range back stress (referred to as Type II back stress) that operates over a significantly larger spatial scale (Figure 4d). Studies indicate that this Type II back stress is the dominant contributor to HDI strengthening in heterogeneous materials, accounting for 30–50% of the total yield strength.^66^

In addition to its strengthening role, the HDI mechanism significantly enhances material ductility through strain partitioning and coordinated deformation. The presence of back stress prevents the soft domains from rapidly entering a strain localization phase after the initial yielding. Instead, constrained by back stress, they continue to elevate their flow stress, while the hard domains gradually yield under the influence of tensile stress. This multistage yielding process dynamically shifts plastic flow between soft and hard domains, thereby avoiding excessive strain accumulation in any single region. This temporally staggered strain accommodation extends the overall uniform deformation stage. The interface constraint effect at domain boundaries is also crucial for ductility. As strong barriers, these boundaries promote the pile-up and storage of GNDs, which later act as secondary dislocation sources during advanced deformation stages, supplying dislocations for sustained plastic flow. Gradient structures serve as a representative model system for elucidating the HDI strengthening mechanism.^2,40,67,68^ A continuous gradient from nanocrystalline surface layers to coarse-grained interior regions creates a gradual mechanical mismatch from the hard to soft domains. Strain transfers progressively from the high-strain-capacity coarse-grained regions to the high-strength surface layers, generating a smooth strain gradient across the gradient zone and thereby avoiding strain concentrations (Figure 4e).^69^ Simultaneously, the relatively continuous distribution of GNDs induced by the gradient establishes a stable long-range back stress field, leading to more stable combinations of strength and toughness and sustained work hardening. Consequently, mesoscale deformation coordination, leveraging HDI stress and the spatial partitioning of strain, allows soft and hard domains to alternately assume dominant roles across different deformation stages, jointly constituting a continuous source of both strength and toughening.

Mesoscale deformation coordination not only improves strength-ductility synergy but also confers unique damage tolerance capabilities to materials. This tolerance stems primarily from the blunting effect exerted by heterogeneous microstructures on the crack tips. When microcracks initiate in hard phases or at interfaces, the alternating arrangement of soft and hard domains provides a dual protective mechanism. On one hand, the geometric and mechanical discontinuities at heterogeneous interfaces cause crack paths to deflect or bifurcate, forcing cracks to propagate in a nonplanar manner.^69^ This significantly increases the energy required for crack propagation. On the other hand, when a crack extends into a soft domain, the higher plastic capacity facilitates the formation of an enlarged plastic zone ahead of the crack tip. This zone absorbs the deformation energy, effectively blunting the crack tip. For instance, our recent study on a heterogeneous dual-phase steel demonstrated that, in contrast to the traditional understanding that crack tips primarily emit dislocations, the soft phase ahead of a crack front actively absorbs dislocations, substantially reducing local stress concentration and thereby retarding crack propagation (Figure 4f).^49^

In summary, the heterogeneous assembly of multiscale nanostructures at the mesoscale successfully bridges nanoscale dislocation behavior regulation with macroscale deformation coordination and damage evolution control. This coupling of multiscale mechanisms establishes a novel pathway for designing metallic materials that surpass the conventional strength–ductility trade-off.

## Conclusion and Outlook

This review highlights how advanced manufacturing has driven the integrated design of hierarchical nanostructures in metals, leading to substantial performance gains. By leveraging both top-down and bottom-up processing pathways, these techniques introduce severe nonequilibrium thermal and kinetic conditions during fabrication, which facilitate the formation of high densities of crystalline defects, chemical heterogeneities, and metastable phase structures. These features significantly broaden the accessible structural landscape beyond the limits of the traditional equilibrium phase diagrams. Through coordinated construction of defect networks, phase architectures, and grain-level assemblies across multiple scales, these methods provide powerful routes to enhance the mechanical properties.

## Core Challenges: Predictability, Transferability, and Reliability

A central challenge lies in the inherent complexity and sensitivity of the microstructures produced under far-from-equilibrium conditions. Small variations in process parameters can lead to substantial changes in defect distributions, phase morphologies, and grain structures. Existing theoretical models often fall short in predicting such outcomes with quantitative accuracy, limiting the ability to rationally design microstructures for targeted properties. As a result, many of the structure–property relationships established to date remain qualitative or system-specific, and their applicability across different alloys and manufacturing routes is limited. This hampers the translation of laboratory discoveries into scalable engineering applications.

## Knowledge Gaps in Nanostructure Evolution

The formation of multiscale nanostructures typically involves concurrent thermomechanical processes, including rapid solidification, solute segregation, phase transformations, and dislocation-mediated plasticity. However, the dynamic interplay among these mechanisms and their contributions to the final microstructures remain poorly understood. Most current studies focus on initial and final states with limited insight into the temporal evolution of structure during processing. This lack of mechanistic resolution impedes our ability to predict the long-term performance and stability. Addressing this gap requires advanced in situ characterization tools capable of capturing real-time microstructural evolution across length scales, such as synchrotron X-ray imaging, in situ electron microscopy, and high-resolution neutron scattering as well as multiscale simulation frameworks that connect atomic-level events to macroscopic behavior. The integration of these techniques is essential for developing predictive process-structure models.

## Outlook: Empowered by Data-Assisted Approaches

Looking forward, the integration of data-driven methodologies with advanced manufacturing and materials science presents a promising avenue for accelerating the discovery and optimization of nanostructured metals. High-throughput experimentation and parallelized simulations enable efficient exploration of vast composition-process-structure spaces.^70^ Machine learning models, particularly those guided by physical constraints, can uncover complex, nonlinear correlations among processing parameters, microstructural descriptors, and properties.^71^ These models not only enhance predictive accuracy but also support real-time process control and adaptive manufacturing. The combination of physics-based simulations, such as first-principles calculations, phase-field modeling, and crystal plasticity, with statistical learning techniques will provide a robust framework for quantitative microstructure design. Ultimately, such integrated approaches can transform the development of multiscale materials from empirical trial-and-error into a predictive and scalable design paradigm (Figure 5). By establishing transferable principles that span across alloys and processes, this strategy will facilitate the rational engineering of high-performance nanostructured metals tailored for demanding applications. Data-augmented multiscale design is poised to become a cornerstone of next-generation metallurgical science.

**Figure 5:**
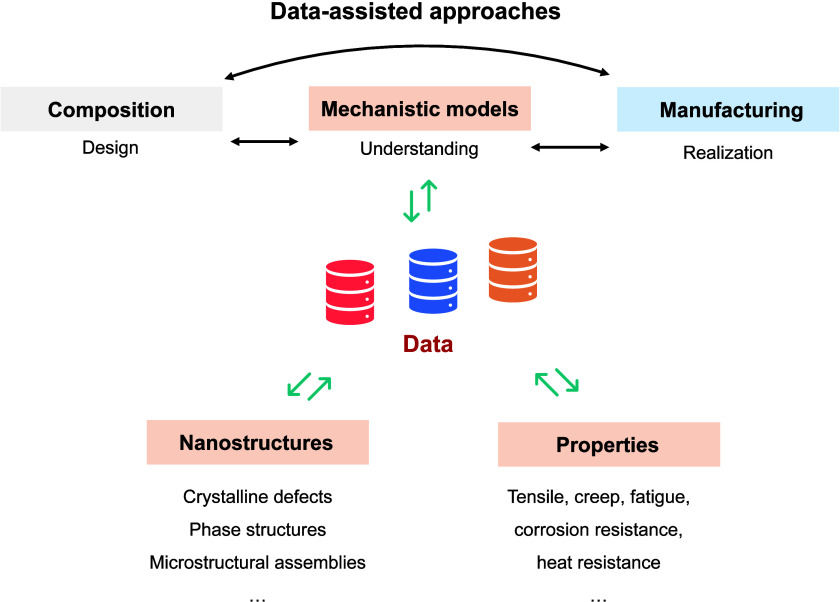
Data-assisted framework for manufacturing-driven multiscale nanostructure–property relationships in metals.
